# Quantification of dose-mortality responses in adult Diptera: Validation using *Ceratitis capitata* and *Drosophila suzukii* responses to spinosad

**DOI:** 10.1371/journal.pone.0210545

**Published:** 2019-02-07

**Authors:** Daniel Valtierra-de-Luis, Maite Villanueva, Javier Caballero, Isabel M. Matas, Trevor Williams, Primitivo Caballero

**Affiliations:** 1 Institute for Multidisciplinary Applied Biology Research (IMAB), Universidad Pública de Navarra, Mutilva, Spain; 2 Centro Interdisciplinar de Investigação Marinha e Ambiental (CIIMAR), Universidade do Porto, Matosinhos, Portugal; 3 Instituto de Ecología AC, Xalapa, Veracruz, Mexico; 4 Departamento de Agronomía, Biotecnología y Alimentación, Universidad Pública de Navarra, Pamplona, Spain; University of Thessaly School of Agricultural Sciences, GREECE

## Abstract

Quantitative laboratory bioassay methods are required to evaluate the toxicity of novel insecticidal compounds for pest control and to determine the presence of resistance traits. We used a radioactive tracer based on ^32^P-ATP to estimate the volume of a droplet ingested by two dipteran pests: *Ceratitis capitata* (Tephritidae) and *Drosophila suzukii* (Drosophilidae). Using blue food dye it was possible to distinguish between individuals that ingested the solution from those that did not. The average volume ingested by *C*. *capitata* adults was 1.968 μl. Females ingested a ~20% greater volume of solution than males. Adults of *D*. *suzukii* ingested an average of 0.879 μl and females ingested ~30% greater volume than males. The droplet feeding method was validated using the naturally-derived insecticide spinosad as the active ingredient (a.i.). For *C*. *capitata*, the concentration-mortality response did not differ between the sexes or among three different batches of insects. Lethal dose values were calculated based on mean ingested volumes. For *C*. *capitata* LD_50_ values were 1.462 and 1.502 ng a.i./insect for males and females, respectively, equivalent to 0.274 and 0.271 ng a.i./mg for males and females, respectively, when sex-specific variation in body weight was considered. Using the same process for *D*. *suzukii*, the LD_50_ value was estimated at 2.927 ng a.i./insect, or 1.994 ng a.i./mg based on a mean body weight of 1.67 mg for both sexes together. We conclude that this technique could be readily employed for determination of the resistance status and dose-mortality responses of insecticidal compounds in many species of pestiferous Diptera.

## Introduction

Simple and repeatable laboratory bioassay methods are required to evaluate the toxicity of novel insecticidal compounds for pest management and to determine the presence of resistance traits in pest populations [1]. The most suitable bioassay methods usually reflect the mode of action of the toxicant and the biology of the pest. Established assay methods therefore use direct topical application, spray droplet delivery systems, such as the Potter tower, or contact with residues on treated surfaces to establish concentration-mortality responses of pests under controlled laboratory conditions [2,3]. These methods have proved to be valuable for compounds that are absorbed following contact with the insect cuticle, such as organophosphates or pyrethroids [4–6]. However, many of the latest generation of insecticidal products that have a selective toxicity spectrum are active by ingestion. Examples include naturally-derived products such as spinosad, avermectins, and *Bacillus thuringiensis* (Bt), and synthetic compounds such as neonicotinoids and diamides, among others [7].

The accurate quantification of toxicity requires the ability to deliver a known quantity of toxicant onto, or into, the pest. For leaf-feeding pests this can be accomplished by placing known quantities of toxicant on leaf discs or on pieces of artificial diet that, when consumed, deliver a known dose of toxicant. For flies however, precise delivery of toxicants by ingestion is more challenging as many feed on liquids that are taken into the crop before being gradually released into the midgut for digestion and assimilation [8]. In the case of dipterans, quantification of the concentration-mortality response requires a method for distinguishing flies that have fed on the toxicant solution from those that have not. This can be achieved by mixing the toxicant with an inert food coloring agent that can be visualized through the abdomen of flies that consumed the colored liquid [9]. In contrast, dose-mortality studies require quantification of the volume of toxicant solution ingested by experimental flies.

In the present study we demonstrate how the use of food-dye based differentiation of toxicant-treated individuals and quantification of ingested volume can be used to characterize the dose-mortality relationship according to sex and body weight of two pestiferous invasive species of Diptera. These species were *Ceratitis capitata* (Wiedemann) (Tephritidae) and *Drosophila suzukii* (Matsumura) (Drosophilidae), both of which are recognized as pests that should be subjected to international quarantine regulations [10]. *Ceratitis capitata* is a polyphagous pest affecting more than 250 species of fruits and vegetables [11]. This fly can survive across a wide range of hosts and climatic conditions and has become established in the Mediterranean region, Africa, the Middle East, Latin America and Western Australia [10]. *Drosophila suzukii* is an invasive pest endemic to south-east Asia that has recently established in most European countries, North America, and the Middle East. This pest attacks a wide range of both cultivated and wild soft-skinned fruits, particularly berries [12]. The larval stages of both these species feed deep within fruit, protected from external applications of pesticides. Consequently, control strategies targeted at these pests usually focus on the adult stage through the use of traps, toxic bait stations, or foliar or bait sprays containing insecticides applied to host plant foliage, where adults may be resting or feeding [13–16]. The use of the sterile insect technique may also be effective in some regions [17].

In the present study we use a radioactive tracer to estimate the volume ingested by flies of each sex and species and perform a proof-of-concept study using the naturally-derived insecticide, spinosad to determine concentration-mortality, dose-mortality and dose per mg body weight relationships. Spinosad was selected for this study as it is highly active by ingestion and has proven to be effective in the control of fruit flies in many parts of the world [18,19].

## Materials and methods

### Insect colonies

Pupae of *C*. *capitata* were obtained at weekly intervals from a colony maintained at the Universidad Politécnica de Madrid, Spain. These insects had been reared on an artificial diet as described previously [20], and had no history of exposure to insecticides. Following emergence, adults were held in ventilated plastic cages 11 x 9 x 8.5 cm with continuous access to water and a mixture of sucrose and hydrolyzed brewer’s yeast (4:1) as food. A laboratory colony of *D*. *suzukii* was started using pupae obtained from the Institut de Recerca i Tecnologia Agroalimentàries [IRTA], Barcelona, Spain. The colony was maintained in the Instituto de Agrobiotecnología, Mutilva, Spain, on a solid semi-synthetic diet comprising 10.5g/l agar, 60 g/l brewer’s yeast, 50 g/l sucrose, 10 g/l ground soybean, 60 g/l maize flour, 10 ml/l ethanol, 5 ml/l propionic acid and 20 mM methyl-paraben. Adult flies were maintained in 14 l ventilated plastic containers. Adults had continuous access to water and the same diet as used to rear larvae. Adult flies of both species and all experimental procedures described in the following sections were performed under the same laboratory conditions of 24 ± 1°C, 85 ± 10% relative humidity and a 16 h: 8 h (light: dark) photoperiod.

### Determination of ingested volume by adult flies

Groups of 50 flies of both sexes that had emerged in the previous 24 h period were placed in 300 ml plastic cups with a muslin lid. Flies were starved, without access to food or water, for 12 h and were then given access to 5 μl droplets of a solution comprising radiolabeled adenosine triphosphate (ATP, γ-^32^P, 3000Ci/mmol, PerkinElmer), 0.1 mg/ml blue food dye (Brilliant blue FCF, Hilton Davis, USA), 0.5% (wt./vol.) hydrolyzed protein (Attrack, Cheminova Agro, Spain), 15% (wt./vol.) sucrose. Flies were allowed to consume the liquid during a 20 min period and were then immediately placed in a -20°C freezer for 1 h until completely frozen. The individuals that had consumed the radioactive liquid were identified by the blue coloration of the intestine observed through the abdominal wall. These individuals were sexed and placed individually in a plastic tube (MicroBeta Trilux 4 ml counting vials, PerkinElmer) with 1.5 ml Ultima Gold liquid scintillation cocktail (PerkinElmer). Adult flies with unstained or partially-stained abdomens were discarded. The radioactivity of each intact individual was determined over a 60 second period using a scintillation counter (MicroBeta 1450 Trilux Wallac, Perkin Elmer, USA). Each fly was measured five times and the average value was calculated after correction for background radiation. The average ingested volume value was determined by comparing the average number of counts obtained from each fly with a calibration curve previously determined using a range of dilutions of ATP-γ-^32^P (Supplemental material, S1 Fig). The experiment was performed using a total of 112 *C*. *capitata* adults (57 female, 55 male) and 64 *D*. *suzukii* adults (42 female, 22 male).

### Body weight of flies

To determine the body weight of flies, adults that had emerged 12–24 h previously and had not mated, were placed in a 300 ml plastic cup and starved for 12 h. Cups were then placed in an Anaerocult A anaerobic jar (Merck, Germany) with dry ice to generate an anaerobic atmosphere that anaesthetized flies. After 15 minutes, each fly was weighed individually to a precision of 0.1 mg using an electronic balance (Ohaus Pioneer, USA). A total of 40 *C*. *capitata* and 30 *D*. *suzukii* individuals of each sex were weighed.

### Validation of method using spinosad

As a proof-of-concept, toxicity assays were performed on both species of flies. For bioassays with *C*. *capitata*, 15–20 adults were collected in 300 ml plastic cups sealed with a muslin lid and starved for 12 h. Flies were then offered 5 μl droplets placed on a piece of parafilm for 20 min (30 droplets in total, ~5 mm distance between droplets). Experimental droplets contained 0.1 mg/ml blue food dye, 0.5% (wt./vol.) hydrolyzed protein (Attrack 300, Cheminova Agro, Spain), 15% (wt./vol.) sucrose and spinosad as the active ingredient (a.i.). The concentration-mortality response was determined in groups of 15–20 adults of both sexes that consumed one of six different concentrations of spinosad (Spintor 480SC, Dow AgroScience, Spain) from 0.2 to 1.5 μg a.i./ml (previously estimated to kill between 5 and 95% of experimental insects). In the case of *C*. *capitata* males, the range of concentrations was 0.3 to 1.5 μg a.i./ml. Droplets of an identical solution containing distilled water alone were offered to insects as a control. After 20 min, the parafilm strip with droplets was removed and adult flies that did not have fully blue-colored abdomens were removed and discarded, leaving approximately 20 flies in each 300 ml cup. A 25 ml plastic cup containing a small piece of sponge soaked with 20 ml of a liquid diet comprising 30% sucrose, 0.5% Attrack and 0.05% methyl-paraben as preservative, was then placed into the larger cup. Mortality was recorded at 5 days post-treatment, by which time LC_50_ values had plateaued according to a previous study ([18], Supplemental material S2 Fig). A total of four batches of *C*. *capitata* were tested. The assay was replicated four times for batches 1 and 2 and three times for batches 3 and 4. Males and females were assayed separately in batch 4.

Bioassays with *D*. *suzukii* adults were performed using a similar design except that droplets of spinosad solution were 3 μl rather than 5 μl in volume and adult diet contained 5% (wt./vol.) soy peptone (Conda, Spain) instead of 0.5% Attrack. The concentration-mortality response was determined using six concentrations of spinosad ranging from 1.39 μg/ml to 5.2 μg/ml. This assay was performed on three occasions using three different groups of insects (replicates) from the laboratory colony.

### Statistical analyses

Ingested volumes and body weights of adult flies of each sex were compared by Welch’s unequal variances t-test. Concentration-mortality data were subjected to logit regression to estimate the median lethal concentration (LC_50_). Abbott’s correction was applied to mortality data prior to analysis to correct for low levels of control mortality [21]. The significance of treatment and interaction terms was determined by sequential removal of terms from the complete logit regression model. All statistical procedures were performed using R software (v. 3.5.1).

## Results

### Determination of ingested volume by adult flies

**Fig 1:**
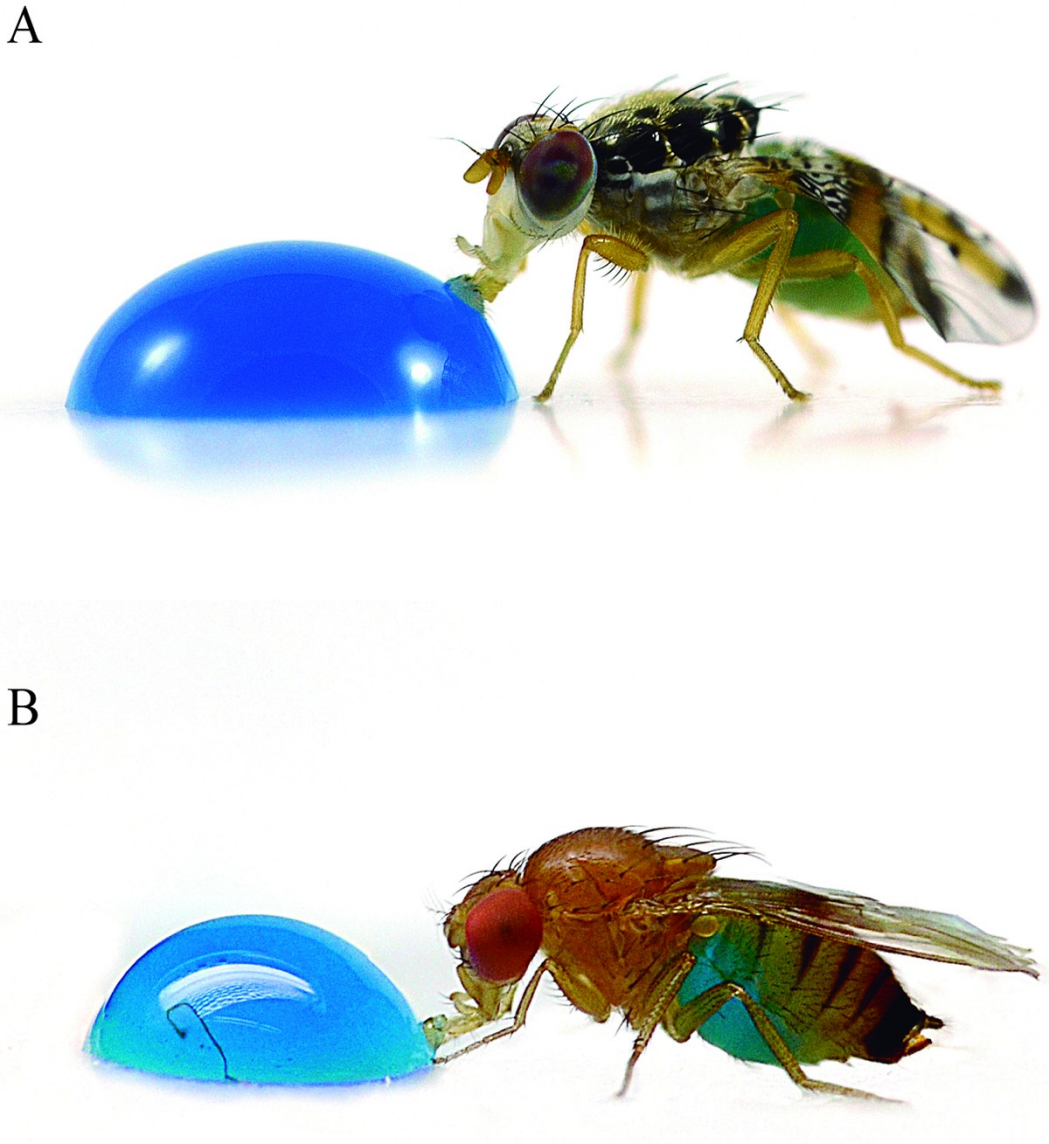
Adults of (A) *Ceratitis capitata* and (B) *Drosophila suzukii* that fed on experimental droplets could be identified by the blue coloration of their abdomen.

Using the blue dye in the ingested experimental solution it was possible to differentiate individuals of *C*. *capitata* and *D*. *suzukii* that ingested the solution from those that did not ingest it, or those that partially ingested it (Fig 1A and 1B). Insects that did not consume the solution in the 20 min feeding period were discarded.

The average (±SE) volume of solution ingested by *C*. *capitata* adults was 1.968 ± 0.049 μl (Table 1), but this differed significantly between sexes with females ingesting ~20% greater volume of solution than males (Welch t = 4.96, d.f. = 94.8, P<0.001). In contrast, adults of *D*. *suzukii* ingested an average of 0.879 ± 0.035 μl and females ingested ~30% greater volume than males (Welch t = 4.69, d.f. = 50.07, P<0.001) (Table 1, S1 Fig).

**Table 1:** Mean ingested volume (±SE) per individual for each sex separately and both sexes of *Ceratitis capitata* and *Drosophila suzukii* adults.

Species	Sex	Number of individuals	Ingested volume ± SE (μl/fly)
***C*. *capitata***	Both sexes	112	1.968 ± 0.049
	Male	55	1.741 ± 0.075
	Female	57	2.187 ± 0.050
***D*. *suzukii***	Both sexes	64	0.879 ± 0.035
	Male	22	0.692 ± 0.046
	Female	42	0.977 ± 0.040

### Body weight of flies

The average (±SE) live weight of individual *C*. *capitata* was 5.43 ± 0.06 mg. The average weight of males (5.33 ± 0.09 mg) was not significantly different from that of females (5.54 ± 0.09 mg) (Welch t = 1.61, d.f. = 79.78, P = 0.110). In contrast, the average live weight of *D*. *suzukii* adults was 1.67 ± 0.09 mg and females (2.063 ± 0.055 mg) were significantly heavier than males (1.30 ± 0.05 mg) (Welch t = 10.61, d.f. = 56.87, P<0.001).

### Validation of method using spinosad

Mortality in control groups of insects ranged from 0–4% and this was used to adjust observed mortality using Abbott's correction [21]. In all cases, mortality increased with increasing concentration of spinosad. For *C*. *capitata*, the LC_50_ values varied between 0.677 and 0.773 μg a.i./ml for both sexes together (Table 2). The concentration-mortality response was similar among batches of insects (batch: χ^2^ = 1.06, d.f. = 2, P = 0.588), although the slope of the response differed significantly among batches 1–3 (interaction batch*concentration: χ^2^ = 25.7, d.f. = 12, P < 0.001). When sexes were treated separately (batch 4), estimated LC_50_ values were 0.840 μg a.i./ml in females and 0.687 μg a.i./ml in males, and the concentration-mortality response did not differ significantly between the sexes (sex: F_1,9_ = 0.028, P = 0.872; interaction sex*concentration: F_1,7_ = 2.68, P = 0.146) (Table 2).

**Table 2:** Logit regression of concentration-mortality response of *Ceratitis capitata* and *Drosophila suzukii* adults that consumed a range of concentrations of spinosad. Bioassays were performed on three batches of *C*. *capitata* (both sexes) and an additional batch (4) in which each sex was treated separately.

Batch	Regression		LC_50_	95% C.I.		LD_50_	95% C.I.	
	Slope ± SE	Intercept ± SE	μg a.i. /ml	Lower	Upper	ng a.i. /insect	Lower	Upper
*Ceratitis capitata*								
1	2.84 ± 0.29	-2.19 ± 0.22	0.773	0.665	0.881	1.521	1.308	1.733
2	4.45 ± 0.40	-3.22 ± 0.28	0.723	0.640	0.806	1.423	1.259	1.586
3	5.72 ± 0.62	-3.87 ± 0.40	0.677	0.597	0.757	1.332	1.175	1.490
4 (♂)	4.05 ± 0.39	-3.39 ± 0.39	0.840	0.726	0.948	1.462	1.263	1.650
4 (♀)	5.17 ± 0.56	-3.55 ± 0.38	0.687	0.597	0.777	1.502	1.305	1.699
*Drosophila suzukii*								
1	1.42 ± 0.14	-4.72 ± 0.46	3.330	3.223	3.437	2.927	2.799	3.336

LD_50_ values were calculated based on LC_50_ values and the volume of solution ingested on average by each species (shown in Table 1).

When the mean volume of toxicant solution consumed by insects was considered, LD_50_ values for *C*. *capitata* varied from 1.332 to 1.521 ng a.i./insect for both sexes together, compared to 1.462 and 1.502 ng a.i./insect for males and females, respectively (Table 2). When the mean of body weight of *C*. *capitata* adults was taken into account, LD_50_ values per mg of insect body weight were 0.280, 0.261, 0.245 ng a.i./mg for both sexes together in batches 1–3, respectively, and were 0.274 and 0.271 ng a.i./mg for males and females in batch 4, respectively.

Using the same process, for *D*. *suzukii* the LC_50_ value was estimated at 3.330 μg a.i./ml and the LD_50_ value was estimated at 2.927 ng a.i./insect, based on the mean ingested volume of 0.879 ± 0.035 μl/insect (Table 1). Similarly, for *D*. *suzukii* the LD_50_ value per mg of live body weight was 1.994 ng a.i./mg based on a mean body weight of 1.67 ± 0.09 mg for both sexes together.

## Discussion

Quantification of ingestion of a sucrose solution containing ^32^P-labeled ATP revealed that *C*. *capitata* males and females ingested an average of 1.74 and 2.19 μl of solution respectively, whereas *D*. *suzukii* consumed 0.69 and 0.98 μl for each sex, respectively (Table 1). This information was used to estimate lethal dose response of both species to the naturally derived insecticide spinosad, from lethal concentration response studies. The 50% lethal dose (LD_50_) of *C*. *capitata* to spinosad was approximately half that of *D*. *suzukii*, although in terms of live body weight, *C*. *capitata* was approximately 7-fold more susceptible to spinosad than *D*. *suzukii* (based on values of 0.27 and 1.84 ng a.i./mg body weight, respectively).

Spinosad has been used in bait formulations to control tephritid pests for almost two decades [19,22], whereas its use against *D*. *suzukii* [23,24] has largely coincided with the global expansion of this pest and its introduction into North America and Europe over the past decade [25]. This compound has a highly favorable ecotoxicological profile and has a low impact on most insect natural enemy populations [26,27].

Previous estimates of spinosad toxicity to *C*. *capitata* have ranged from 0.896 mg a.i./l at 48 h post-treatment [28] to 0.24–0.28 mg a.i./l following five days of continuous exposure [19,29], whereas studies involving a different methodology reported significantly higher LC_50_ values of 2.8 and 4.2 mg a.i./l for males and females, respectively, at 24 h post-treatment [27]. Individuals of *C*. *capitata* that survive exposure to sublethal quantities of spinosad can also experience negative effects on fecundity, egg fertility, and adult longevity, in addition to sex-dependent effects on the expression of a number of immune-modulating genes [29].

Previous studies on spinosad toxicity to *D*. *suzukii* reported that high concentrations resulted in 100% mortality, although concentration-mortality responses were not determined [15,24,30–32]. One exception involved a study on spinosad contact toxicity to two *D*. *suzukii* populations in which contact LC_50_ values of 2.78 and 7.60 mg a.i./l were estimated following 6 h of exposure [33].

In addition to the dose of toxicant consumed, the influence of speed of kill of the compound can affect the apparent toxicity of a compound in laboratory assays, in which measures of mortality are taken shortly after exposure to the toxicant. In the present study, we adopted a 5-day period for assessment of spinosad-induced mortality, based on a previous study in which spinosad-induced cumulative mortality increased and estimated LC_50_ values plateaued over a 7-day period ([18], S2 Fig). This reflected the slower speed of kill of spinosad compared to fast acting compounds, such as pyrethroids. However, the 5-day period of post-treatment monitoring did not adversely affect the prevalence of mortality of untreated control insects that never exceeded 4% mortality in any case.

Females of both species consumed larger volumes of toxicant solution than conspecific males, although when lethal dose per mg of body weight was calculated, the susceptibility of both sexes to spinosad was similar in *C*. *capitata* (0.27 ng a.i./mg body weight for both sexes). In contrast, in *D*. *suzukii* the sexes were not treated separately as this would involve chilling or anaesthetizing them for manual sorting into sexes, which given their small size may have affected their survival during the post-treatment period.

Quantification of ingestion by dipterans is an issue that has received considerable attention, mainly because *D*. *melanogaster* has become a laboratory model for studies on physiology, nutrition and longevity for which precise estimates of feeding rates are required [34]. The quantitative methods developed to date include the use of calibrated capillary feeding tubes (the CAFE system) [35], recording the proboscis extension feeding response [34,36], and the use of food in droplets mixed with dye [37] or radioactive tracer compounds [38,39]. Each of these methods has advantages and drawbacks, but for studies over short periods, the use of radiolabeled tracers in droplets of food has been shown to be highly sensitive, consistent and compatible with natural feeding behavior in *Drosophila* [40]. This aspect of feeding on droplets is likely to extend to other dipterans such as tephritids that feed on droplets of honeydew, fruit juices or bird droppings in the natural environment [41–43]. From a logistical standpoint, once calibrated, the droplet-feeding assay is also quick and easy to set up and the presence of a visible dye in the mixture allows individuals that feed on toxicant droplets to be readily distinguished from those that did not. The food dye based methodology, either as is or slightly modified, could also be used to study the deterrence of fruit flies to ingestion of insecticides by assessing the prevalence of flies with unstained or partially-stained abdomens.

There are numerous sources of variation in insect bioassays involving ingestion of toxins or pathogens that have to be managed to control overdispersion in the results [44,45]. In the case of dipterans, these include individual-level effects, such as sporadic or irregular feeding patterns over time, variation in meal size, and body size, developmental and physiological effects. Genetically distinct strains of insects are also expected to feed and respond differently to a given inoculum or toxicant [44,46], particularly if they have a history of exposure to a given compound [47]. Moreover, as apparent in the present study, there is often sex-dependent variation in insect ingestion and sensitivity to toxicants. In the case of *D*. *melanogaster*, females that have mated also show a marked increase in feeding, probably in a response to the energetic requirements for egg development [48].

Much of the variation present in insect bioassays can be controlled by standardizing insect rearing conditions (diet, density, temperature, etc.), selecting individuals of the same age and developmental stage, limiting the time available for ingestion of the toxicant to avoid repeated feeding events and using visible dyes to identify individuals that have not engaged in feeding on toxicant droplets. These were precisely the steps that we adopted to minimize variation in our bioassays.

The period during which experimental droplets can be ingested, 20 min in the case of our study, is relevant as excretion begins approximately 40 mins after feeding in *Drosophila* [34], which would have adversely affected the quantification of ingested volumes in the radioactive tracer study. Fortunately, the steps that we took to standardize experimental insect selection and dosing procedures resulted in low levels of variation within assays and among assays involving different batches of insects in the case of *C*. *capitata* (Table 2). This type of droplet feeding assay can be readily calibrated to quantify the susceptibility of other species of pestiferous Diptera to insecticidal compounds or pathogens that act by ingestion, such as *Bacillus thuringiensis* or some viruses [49–53].

We conclude that the present study represents a quantitative method for determining the toxic properties of insecticidal compounds or pathogens that act by ingestion in adult Diptera that feed by consuming droplets. The method is simple and reproducible. To our knowledge, this is also the first report of quantification of lethal dose responses by ingestion of an insecticide and ingested lethal doses per mg of insect body weight in adult Diptera.

## Supporting information

S1 Fig Calibration curve for quantification of ingested volume in (A) *Ceratitis capitata* and (B) *Drosophila suzukii*.
(DOCX)

S2 Fig Changes in 50% lethal concentration (LC_50_, blue points) and 90% lethal concentration (LC_90_, orange points) values at different intervals (0.5–7 days) following ingestion of spinosad by *Ceratitis capitata* adults, as reported by Adan et al. [18]. (DOCX)

S1 File An Excel file comprising five worksheets with raw data. (XLSX)
