# Supplementary material for: Quantification of dose-mortality responses in adult Diptera: Validation using Ceratitis capitata and Drosophila suzukii responses to spinosad
Source: PLoS One. 2019 Feb 7;14(2):e0210545. doi: 10.1371/journal.pone.0210545 (PMC6366873; doi:10.1371/journal.pone.0210545)

**S1 Fig.** **Calibration curve for quantification of ingested volume in (A) *Ceratitis capitata* and (B)** ***Drosophila suzukii.*** Adult flies ingested experimental droplets containing 32P -labelled adenosine triphosphate (3000Ci/mmol, PerkinElmer), in mixtures with fluorella blue, hydrolyzed protein and sucrose (see Methods section of text). Red dots indicate the average of five 1-min counts in a scintillation counter plotted against a range of dilutions of ATP-γ-^32^P. The resulting correlation lines and equations are shown.


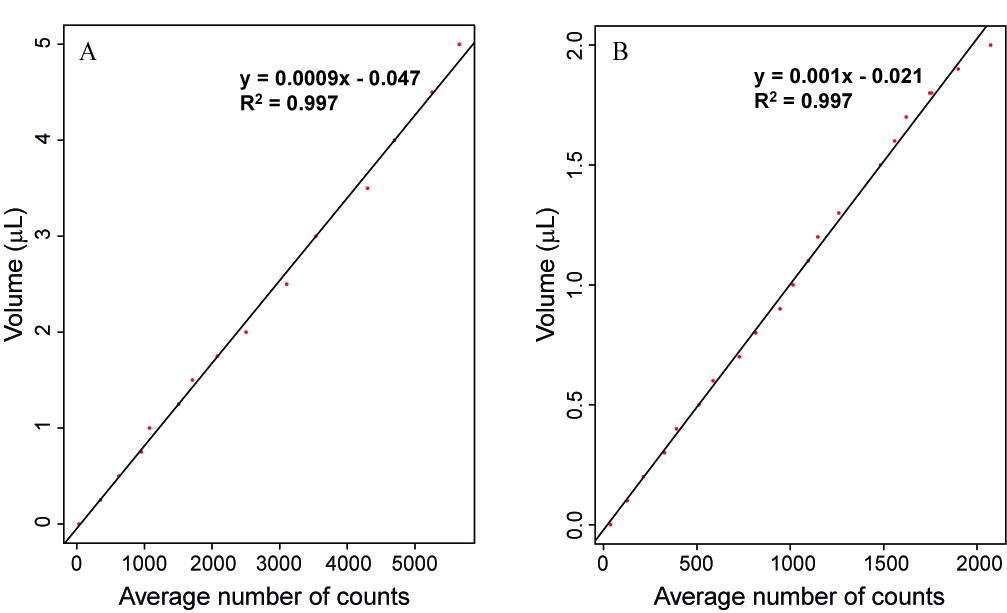

Supplement: S1 Fig — Calibration curve for quantification of ingested volume in (A) Ceratitis capitata and (B) Drosophila suzukii. (DOCX) [file pone.0210545.s001.docx]
