# Supplementary material for: Quantification of dose-mortality responses in adult Diptera: Validation using Ceratitis capitata and Drosophila suzukii responses to spinosad
Source: PLoS One. 2019 Feb 7;14(2):e0210545. doi: 10.1371/journal.pone.0210545 (PMC6366873; doi:10.1371/journal.pone.0210545)
Supplement: S2 Fig — (DOCX) [file pone.0210545.s002.docx]

**Supplemental material**

**S2 Fig.** **Relationship between LC_50_ (blue points) and LC_90_ (orange points) values in mg active ingredient (a.i.)/liter at different intervals (0.5 - 7 days) following ingestion of spinosad by *Ceratitis capitata* adults, as reported by Adan et al. (1995).** Vertical bars are asymetrical and indicate 95% confidence intervals of estimated value.


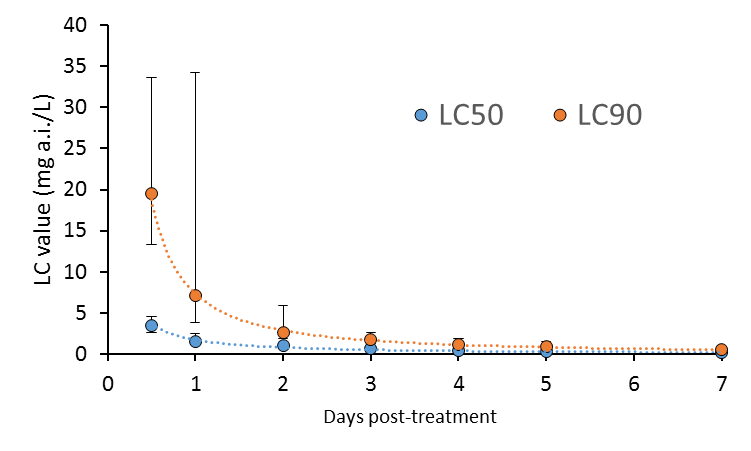


Source: Adán, A., Estal, P.D., Budia, F., González, M., Viñuela, E., 1996. Laboratory evaluation of the novel naturally derived compound spinosad against *Ceratitis capitata*. Pesticide Science 48, 261-268.
